# Population pharmacokinetics of tofacitinib in patients with active ankylosing spondylitis 

**DOI:** 10.5414/CP204781

**Published:** 2025-12-08

**Authors:** Shinichi Tsuchiwata, Akiyuki Suzuki, Qiang Wang, Keith Kanik, Lara Fallon, Sujatha Menon

**Affiliations:** 1 Pfizer R&D Japan, G.K., Tokyo, Japan,; 2 Pfizer Inc, Groton, CT, USA, and; 3 Pfizer Canada, Kirkland, Quebec, Canada

**Keywords:** ankylosing spondylitis, population pharmacokinetics, tofacitinib

## Abstract

Objective: To characterize tofacitinib pharmacokinetics (PK) in patients with active ankylosing spondylitis (AS) and estimate the effects of covariates on variability of PK parameters. Materials and methods: Pooled data from two studies in patients with AS who received tofacitinib were analyzed using nonlinear mixed-effects modeling. Tofacitinib PK was described by a one-compartment model parameterized in terms of apparent oral clearance (CL/F), apparent volume of distribution (V/F), and a first-order absorption rate constant (k_a_). Covariates evaluated: baseline age, sex, race, creatinine clearance (BCCL), and C-reactive protein for CL/F; baseline age/body weight for V/F. Results: Analysis included 279 patients. The point estimates for CL/F, V/F, and k_a_ were 27.1 L/hour, 126 L, and 3.07 hour^–1^, respectively, in a reference patient. Excluding BCCL, point estimates of area under the concentration–time curve (AUC) over a dosing interval at steady-state and maximum steady-state tofacitinib concentration (C_max_) change vs. the reference patient ranged from 98 – 112% and 89 – 115%, respectively. Estimated AUC was 24% higher in a patient with BCCL = 50 mL/min vs. the reference patient (BCCL = 126 mL/min). Point estimates and 90% confidence intervals of the AUC and C_max_ ratios indicated no major differences in tofacitinib exposure over the range of baseline age/body weight studied, and sex/race. Conclusion: Tofacitinib does not require dose adjustment/restriction for age, body weight, sex, or race based on the differences (< 20%) in exposure relative to a reference patient with AS. The tofacitinib CL/F and BCCL relationship was consistent with known contribution of renal excretion to total tofacitinib clearance.

Clinical trial registration: NCT01786668; NCT03502616

**What is known about this subject**

- Tofacitinib is an oral Janus kinase inhibitor for the treatment of ankylosing spondylitis (AS).
- The pharmacokinetics of tofacitinib in healthy volunteers are characterized by rapid absorption and elimination.

**What this study adds**

- This study confirmed that tofacitinib does not require dose modification or restrictions for age, body weight, sex, or race in adult patients with AS, based on < 20% differences in area under the concentration–time curve and maximum steady-state tofacitinib concentration ratios across these covariates relative to a reference patient with AS.

## Introduction 

Ankylosing spondylitis (AS), a subset of radiographic axial spondyloarthritis [1], is a chronic, inflammatory rheumatic disease that can lead to serious impairment of spinal mobility and reduced quality of life [2, 3, 4]. The prevalence of AS has been reported to range from 130 to 1,400 per 100,000 individuals [5]. Non-steroidal anti-inflammatory drugs are the recommended first-line treatment for AS, followed by biologic disease-modifying antirheumatic drugs (bDMARDs), such as tumor necrosis factor inhibitors or interleukin (IL)-17 antagonists [6, 7]. Patients with active AS despite treatment with a bDMARD should consider switching to a Janus kinase (JAK) inhibitor [6, 7]. 

JAK inhibitors directly bind to and modulate the intracellular catalytic activity of JAKs, which are essential enzymes in signaling pathways that mediate cytokine signaling for numerous innate and adaptive immune responses [8, 9]. These include signaling via IL-17, IL-12, and IL-23, which have been implicated in AS pathogenesis [10, 11]. The inhibition of JAKs may therefore suppress articular and extra-articular symptoms of AS [12]. Tofacitinib is an oral JAK inhibitor for the treatment of AS [13, 14]. The efficacy and safety of tofacitinib 5 mg twice daily (BID) have been demonstrated in one phase 2 [15] and one phase 3 [16] randomized controlled trial (RCT). At week 16, a significantly greater proportion of tofacitinib-treated patients achieved Assessment of SpondyloArthritis International Society ≥ 20% improvement or ≥ 40% improvement, compared with placebo-treated patients [16]. No new potential safety risks for tofacitinib were identified in this patient population [16]. 

The pharmacokinetics (PK) of tofacitinib in healthy volunteers are characterized by rapid absorption and elimination, with a time to reach maximum concentration of ~ 0.5 – 1.1 hours and a half-life of ~ 3 hours after immediate release of tofacitinib [17, 18]. The majority of tofacitinib clearance (~ 70%) is via hepatic metabolism (primarily via cytochrome P450 3A4), while only ~ 30% of the clearance is via renal elimination [17]. In general, systemic exposure of tofacitinib increases in a dose-proportional manner regardless of duration or population [17]. 

The characterization of the PK of an agent in the intended disease population provides important insights into the disposition of the agent for the specific patient group. In addition, it measures and explains PK variability from various sources, and allows for further evaluation of the relationships between systemic exposure and pharmacodynamic response, thus informing drug development and regulatory decision making. To date, population PK analyses for tofacitinib have been carried out in patients with rheumatoid arthritis (RA) [19], psoriatic arthritis (PsA) [20], psoriasis (PsO) [21], ulcerative colitis (UC) [22], and juvenile idiopathic arthritis [23]. 

Here, we report a population PK analysis of tofacitinib in patients with AS. We aimed to characterize the PK of tofacitinib in patients with AS, and to identify intrinsic and extrinsic factors (covariates) that impact the PK of tofacitinib in these patients. 

## Materials and methods 

### Study designs and data analysis 

This population PK analysis used pooled data from two double-blind RCTs of tofacitinib in patients with active AS. Tofacitinib-treated patients received doses of 2, 5, or 10 mg BID in a phase 2 dose-ranging study (NCT01786668) [15] or 5 mg BID in a phase 3 study (NCT03502616) [16]; details on study design and patient populations have been published previously. Briefly, in both studies, patients ≥ 18 years of age diagnosed with AS according to the modified New York criteria for AS, and with a Bath AS Disease Activity Index (BASDAI) score of ≥ 4 and back pain score (BASDAI question 2) of ≥ 4, received placebo or tofacitinib for 12 weeks followed by a 4-week washout [15] or for 16 weeks [16]. Tofacitinib plasma concentrations were measured using a previously described, fully validated, quantitative, high-performance liquid chromatography-tandem mass spectrometry assay [24]; assay range for AS samples: 0.1 – 100 ng/mL. For both studies, measurements were taken from sampling at week 4 (pre-dose (planned to occur within 12 ± 2 hours of previous dose of tofacitinib)), and 0.5 and 2 hours post-dose), week 8 (pre-dose, and 0.5, 2, and 3 hours post-dose), and at early termination if applicable (phase 2 study only). Missing tofacitinib concentrations were excluded from the population PK analysis. 

The population PK analysis was conducted using the nonlinear mixed-effects modeling approach. The software packages used were NONMEM version 7.4.3 (ICON plc. Gaithersburg, MD, USA) and Perl-speaks-NONMEM version 4.9.0 as supporting software for the execution of NONMEM. R version 3.6.1 (R Foundation for Statistical Computing, Vienna, Austria) was used for data handling, exploratory data analysis, and creation of graphs for presentations and reports. The estimation method was the first-order conditional estimation method with interaction (FOCEI). 

The analysis was conducted in the following steps: 1) base structural model development, 2) random-effects model development, 3) final full model development, 4) assessment of model adequacy (goodness-of-fit), and 5) model predictive performance (validation). 

### Base structural model and random-effects model development 

A PK model that was previously built for the RA, PsO, and PsA patient populations was used as a starting point for model development [13, 20, 21]. 

Tofacitinib PK was described by a one-compartment disposition model, parameterized in terms of apparent oral clearance (CL/F), apparent volume of distribution (V/F), and a first-order absorption rate constant (k_a_). Inter-individual variability (IIV) on CL/F and V/F were modeled using exponential variance models with OMEGA BLOCK. Additionally, inter-occasional variability was taken into consideration in the parameter F. 

Residual random effects were described with two proportional error models for trough (time after dose ≥ 9 hours) and non-trough (time after dose < 9 hours) data; 9 hours was selected based on the observed profile of tofacitinib concentrations over time to separate them as trough and non-trough values. 

### Final full model development 

Predefined covariates and covariate parameter relationships were identified based on prior information of tofacitinib PK in other patient populations [19, 20, 21, 22] as well as exploratory graphics and scientific interest. Baseline age, sex, race, creatine clearance (BCCL; calculated from Cockroft-Gault equation), and C-reactive protein (BCRP) were evaluated as potential predictors of CL/F, and baseline age and body weight were evaluated as predictors of V/F. 

Body weight and ethnicity (Hispanic or non-Hispanic) were also predefined as potential predictors of CL/F. However, in order to avoid collinearity in predictors, the effect of body weight on CL/F was not employed in the final full model as body weight was correlated with BCCL (correlation coefficient > 0.5). As the majority of patients in the dataset were non-Hispanic/Latino (97.8%), ethnicity was not included for the final full model. A final full model was constructed to avoid correlation or collinearity in predictors. Continuous covariates were incorporated as power functions, normalized to the reference (approximate median) values. Each category of categorical covariates (sex and race) was entered into the model as one coefficient. Population parameters, including fixed-effects and random-effects parameters, were estimated using FOCEI. Goodness-of-fit of different models to the data was evaluated using change in objective function value, visual inspection of various diagnostic plots, and precision of the parameter estimates. 

At all stages of model development, diagnostic plots were examined to assess model adequacy, possible lack of fit, or violation of modeling assumptions. Along with residual plots, shrinkage and precision of parameter estimates were utilized to assess model adequacy. η histogram plots were used to assess the assumption of normality and the appropriateness of the selected parameter variability. Nonparametric bootstrap resampling (with 1,000 replicate datasets) was used to obtain the final precision of the parameter estimates. 

Visual predictive checks (VPCs) were performed for the final base and full PK models. VPCs were stratified by predictors considered important (e.g., study, dose level, body weight, sex etc.). The quality of final full model predictions was determined by the agreement between the intervals of simulated percentiles and estimated percentiles of the observed data. 

### Impact of covariates in the tofacitinib population PK model 

The impact of covariates on CL/F was assessed in patients who were 64 years of age (95^th^ percentile), female, Asian, with body weights of 54 and 107 kg (5^th^ and 95^th^ percentiles, respectively), with a BCCL of 50 mL/min, and with a BCRP of 8 mg/dL, compared with reference patients (40 years of age, male, White, body weight of 78 kg, BCCL of 126 mL/min, and BCRP of 0.851 mg/dL). In addition, the impact of covariates on V/F was assessed in patients who were 64 years of age, and with body weights of 54 and 107 kg compared with reference patients. 

Point estimates for area under the concentration–time curve (AUC) and maximum steady-state tofacitinib concentration (C_max_) were generated using the individual parameters obtained using the population PK model. Confidence intervals (CIs) were estimated from the 1,000 nonparametric bootstrap runs. 

## Results 

### Patient demographics 

The pooled PK dataset consisted of 1,917 tofacitinib plasma concentration measurements from 279 tofacitinib-treated patients (57 females, 222 males; 147 patients from the phase 2 study (2 mg BID, n = 50; 5 mg BID, n = 49; 10 mg BID, n = 48) and 132 (all receiving 5 mg BID) from the phase 3 study)). Patient demographics and baseline characteristics are summarized in Table 1. Approximately 1% of the total observations were reported as data records below limit of quantification and treated as missing and therefore not included in the population PK analysis. 

### Base structural model 

The selected base structural model (a one-compartment disposition model with first-order absorption) adequately described the plasma concentration-time profile of tofacitinib with reasonable or unbiased parameter estimates, in patients with active AS. 

The typical estimate of CL/F was 26.7 L/hour with an IIV of 30.5%, V/F was 124 L with an IIV of 39.2%; the relative standard errors (RSEs) for CL/F and V/F were both < 3%. The k_a_ was estimated as 3.06 hour^–1^ with an RSE of 10.1%. Residual variability for observations with trough and non-trough were 69.5% and 60.3%, respectively. Shrinkage estimates from the base structural model were 21.5% for IIV of CL/F, 24.8% for IIV of V/F, and 8.9% for IIV of residual error. 

### Final full model 

Predefined covariates were introduced to CL/F and V/F in the base structural model to generate a final full model. 

The absolute value of correlation coefficients between explored covariates was < 0.5, except for the correlation between body weight and BCCL, which was 0.58. As BCCL was calculated using the Cockcroft-Gault equation based on patients’ serum creatinine levels as well as age, sex, and body weight, the impact of including both body weight and BCCL on CL/F in the final full model was investigated by testing the models including body weight or BCCL on CL/F. Compared with the final full model with BCCL only, the model with both body weight and BCCL, or with body weight only on CL/F, led to a change in objective function value of –0.066 and +6.301, respectively, indicating that the impact of body weight on CL/F was negligible. 

In addition to body weight, impact of age effect on CL/F was also investigated due to correlation with BCCL, in which the correlation coefficient was –0.412. The reduced model, in which the age effect on CL/F was removed, showed a statistically significant increase of objective function value compared with the final full model (7.980, p < 0.01). The collinearity between BCCL and age was therefore ruled out. Goodness-of-fit plots for the final full model are shown in Supplemental Figure S1. 

The parameter estimates for the final full model are summarized in Table 2. The 95% CIs of the parameters CL/F, V/F, and k_a_, as well as the residual errors, were within narrow ranges, which suggests precise estimation. 

For a typical reference patient (White, male, 78 kg, 40 years old, BCCL 126 mL/min, BCRP 0.851 mg/dL), CL/F was estimated to be 27.1 L/hour, V/F to be 126 L, and k_a_ to be 3.07 hour^–1^ (Table 2). Typical fixed-effects parameters for CL/F, V/F, and k_a_, as well as most of the random-variance parameters, were estimated with good precision. 

Parameter estimates for the effects of covariates in the final full model indicated that the 95% CIs for the effects of BCRP and female sex on CL/F contained the null value. The effects of age, BCCL, and Asian race on CL/F were significant (CIs excluded null). Body weight and age also impacted V/F (CIs excluded null) (Table 2). 

### Model validation: Adequacy and predictive performance 

**Figure 1:**
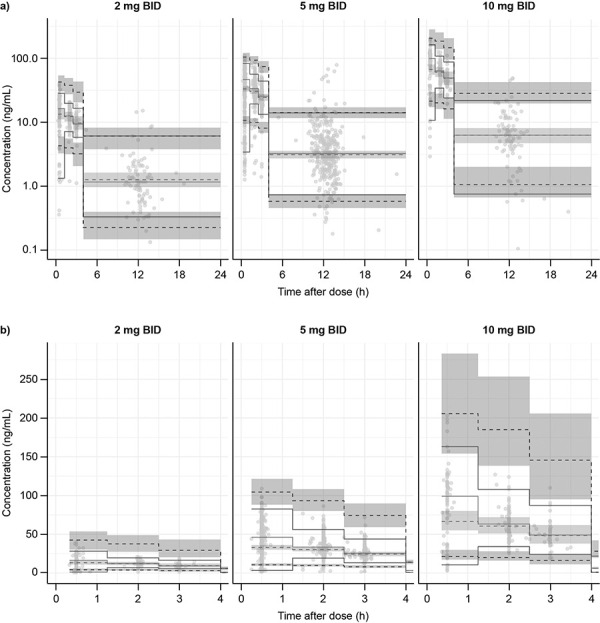
Visual predictive check stratified by dose group for final full model run for A) 24 hours after dose, and B) 4 hours after dose. BID = twice daily; CI = confidence interval; h = hours. For each panel, observed concentration vs. time profile is represented by solid gray lines (50^th^ percentile) and solid black lines (5^th^ and 95^th^ percentiles). Gray dashed lines represent 5^th^ and 95^th^ percentiles. Light gray areas represent 95% CIs of the 50^th^ percentile, with dark gray areas representing the 2.5^th^ and 97.5^th^ percentiles for concentration vs. time profile. Closed circles indicate individual observed concentration-time data.

Model evaluation results, including the results of VPCs, indicated that the final model provided a reliable description of the data with good precision for structural model and variance parameter estimates. The simulated distributions matched the observed concentrations except samples at 0.5 hours (immediately after dosing), which indicated the final full model slightly under predicted the absorption phase (Figure 1). 

### Impact of covariates in the tofacitinib population PK model 

Some inferences could be made using the parameters from the full model: a 64-year-old patient was estimated to have a 10.9% lower CL/F vs. a 40-year-old patient; a female patient was estimated to have a 2.4% higher CL/F vs. a male patient; an Asian patient was estimated to have a 10.3% lower CL/F compared with a non-Asian patient; a patient with a BCCL of 50 mL/min was estimated to have a 19.4% lower CL/F vs. a patient with a BCCL of 126 mL/min (median value in the analysis dataset); and a patient with a BCRP of 8 mg/dL was estimated to have a 4.1% lower CL/F vs. a patient with a BCRP of 0.851 mg/dL. 

A patient of 64 years of age (95^th^ percentile of age) was estimated to have a 10.2% lower V/F vs. a 40-year-old patient. A patient weighing 54 or 107 kg (5^th^ and 95^th^ percentiles of body weight, respectively) was estimated to have a 19% lower or 20% higher V/F vs. a patient with a body weight of 78 kg (median value in dataset), respectively. 

The geometric mean (% coefficient of variation (CV)) values for AUC were 72.2 ng×h/mL (21.8%) for tofacitinib 2 mg BID, 191 ng×h/mL (25.4%) for 5 mg BID, and 372 ng×h/mL (24.7%) for 10 mg BID. The geometric mean (% CV) values for C_max_ were 14.0 ng/mL (24.7%) for tofacitinib 2 mg BID, 36.7 ng/mL (28.0%) for 5 mg BID, and 73.9 ng/mL (22.5%) for 10 mg BID. 

**Figure 2:**
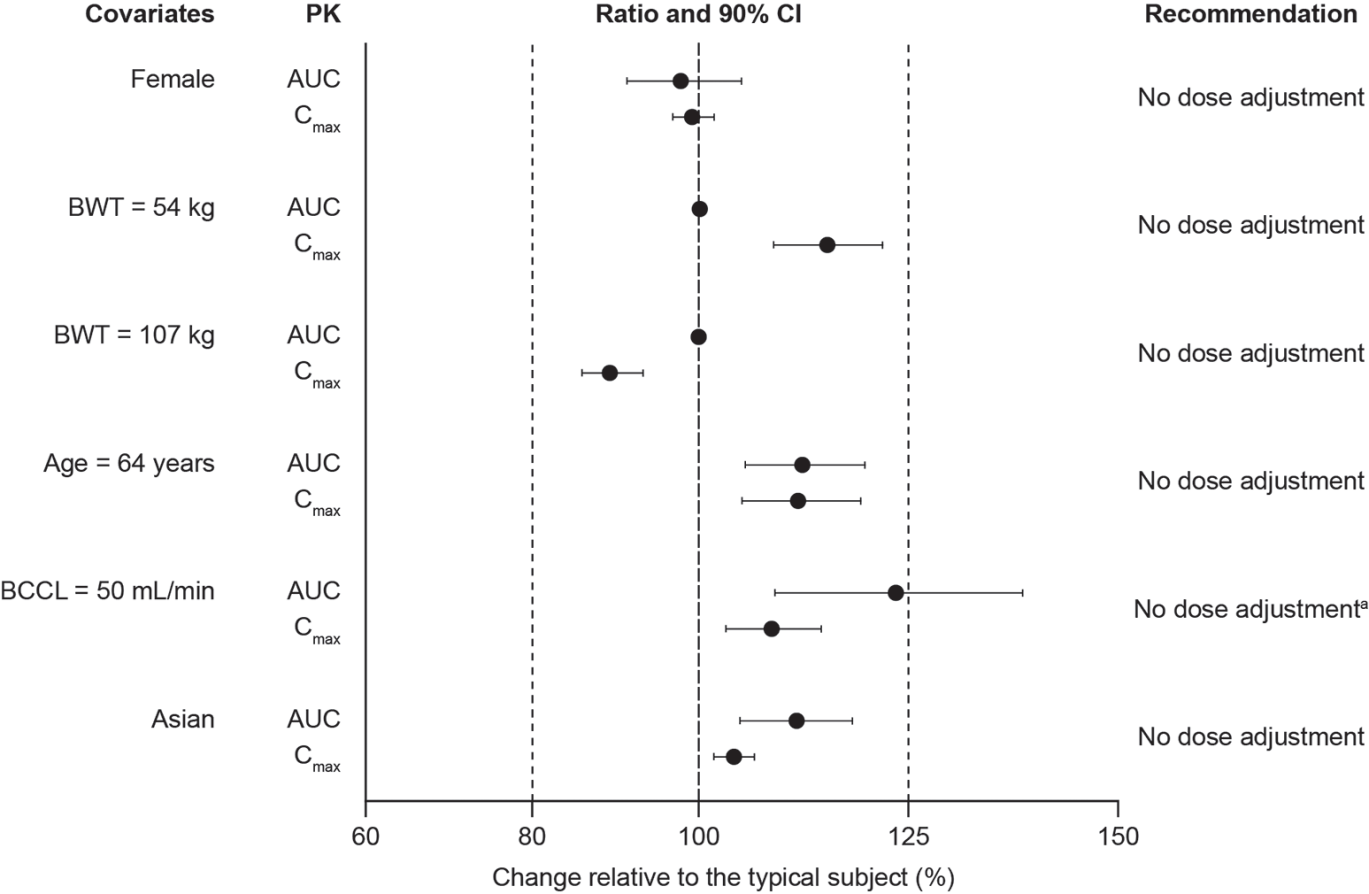
Impact of covariate effects on tofacitinib exposure metrics. AUC = area under the concentration–time curve; BCCL = baseline creatinine clearance; BCRP = baseline C-reactive protein; BWT = body weight; CI = confidence interval; C_max_ = maximum steady-state tofacitinib concentration over the dosing interval; PK = pharmacokinetics. ^a^No dose adjustment was recommended for patients with mild renal impairment in the US prescribing information [13]. Dose adjustment recommendation for BCCL 50 mL/min was based on prior data (phase 1 renal impairment trials), as the dataset for patients with BCCL below 50 mL/min in this analysis was limited (48.1 mL/min was the lowest BCCL in the analysis dataset). The gray dotted line represents limits of a range from 80 – 125%. Magnitude of change is presented in reference to a typical patient (White, male, 78 kg, 40 years old, BCCL 126 mL/min, BCRP 0.851 mg/dL).

The impact of covariate effects on AUC and C_max_ and dose-adjustment recommendations for specified subgroups are shown in Figure 2. With the exception of BCCL, point estimates of AUC and C_max_ change relative to a typical patient ranged between 98 and 112%, and 89 and 115%, respectively (Figure 2). For a patient with a BCCL of 50 mL/min, AUC was estimated to be 24% higher relative to a reference patient with a BCCL of 126 mL/min (Figure 2). As patients with BCCL values below 50 mL/min (as estimated by the Cockcroft-Gault equation) were limited in the pooled PK dataset, the need for dose adjustment in renal impairment was primarily assessed using phase 1 data from studies NCT01740362 and NCT01710020, which evaluated PK in patients with renal impairment and end-stage renal disease, respectively. The point estimates of the AUC and C_max_ ratios, and associated 90% CIs, indicated no major differences (< 20%) in tofacitinib exposure over either the range of ages and body weights studied or race and sex (Figure 2). 

## Discussion 

In this population PK analysis, a one-compartment model, parameterized in terms of CL/F, V/F, and first-order absorption, was found to adequately describe the plasma concentration–time profile of tofacitinib in patients with active AS, which allowed the evaluation of patient-specific covariates on systemic concentrations in these patients. 

Structural and random-effect models were explored in an attempt to better characterize the absorption of tofacitinib in patients with AS. Given the exploratory findings, the most stable model was selected as the base model. Tofacitinib CL/F data in adult patients with AS were generally similar to previously reported CL/F data from PK population analyses of tofacitinib in patients with other immune-mediated inflammatory diseases, such as PsO (26.7 L/h) [25], PsA (31.7 L/h) [20], RA (18.4 L/h) [19], or UC (22.2 L/h) [22], but lower than the clearance estimates from a pooled analysis of healthy individuals [19]. This is not surprising, since it has been previously proposed that lower tofacitinib CL/F in patients with immune-mediated inflammatory disease could be due to downregulation of cytochrome P450 by inflammatory stimuli [26, 27]. The IIV of CL/F in patients with active AS was comparable to the IIVs previously reported in patients with RA [19], PsA [20], or PsO [25]. 

This analysis indicated that patients with a BCCL of 50 mL/min were estimated to have a CL/F that was 19.4% lower than that of reference patients with a BCCL of 126 mL/min (median value in this analysis). These results are consistent with the regression analysis of pooled data from studies of PK in patients with end-stage renal disease and renal impairment (NCT01710020 and NCT01740362, respectively). Based on the < 20% differences in AUC and C_max_ ratios across patient covariates relative to a reference patient with AS, this population PK analysis indicated that tofacitinib does not require dose modifications or restrictions for age, body weight, sex, or race in this patient population. Similar observations have been previously reported for patients with RA or PsA [19, 20]. 

Tofacitinib exposure data in adult patients with AS were similar to previously reported data for tofacitinib in patients with other immune-mediated inflammatory diseases [19, 20]. Plasma exposure for tofacitinib 5 mg BID, as measured by steady-state AUC, was 381 ng×h/mL, which was generally comparable with the AUC values previously reported for RA (504 ng×h/mL), PsA (419 ng×h/mL), and UC (423 ng×h/mL) [13]. 

## Conclusion 

The population PK of tofacitinib in patients with active AS was adequately described by a one-compartment model with first-order absorption. The relationship between tofacitinib CL/F and BCCL is consistent with the known contribution of renal excretion to the total clearance of tofacitinib. Tofacitinib does not require dose modification or restrictions for age, body weight, sex, or race in the adult AS population. 

## Data sharing statement 

Upon request, and subject to review, Pfizer will provide the data that support the findings of this study. Subject to certain criteria, conditions, and exceptions, Pfizer may also provide access to the related individual de-identified participant data. See https://www.pfizer.com/science/clinical-trials/trial-data-and-results for more information. 

## Authors’ contributions 

SM contributed to the study conception. QW, KK, LF, and SM contributed to the study design. QW, KK, and SM contributed to the acquisition of data. ST, AS, QW, and SM contributed to the data analysis. ST, AS, KK, LF, and SM contributed to the data interpretation. All authors contributed to the drafting and critical review of the manuscript content. All authors approved the final manuscript. 

## Funding 

This study was sponsored by Pfizer Inc. Medical writing support, under the direction of the authors, was provided by Karen Thompson, PhD, and Justine Juana, BHSc, of CMC Connect, a division of IPG Health Medical Communications, funded by Pfizer Inc, in accordance with Good Publication Practice (GPP 2022) guidelines (Ann Intern Med 2022; *175:* 1298-1304). 

## Conflict of interest 

ST and AS are shareholders of Pfizer Inc and employees of Pfizer R&D Japan. KK, LF, and SM are shareholders and employees of Pfizer Inc. QW was a shareholder and employee of Pfizer Inc at the time of this analysis. 

**Table 1.** Summary of patient demographics and baseline characteristics.

	Phase 2 study (N = 147)	Phase 3 study (N = 132)	Total (N = 279)
Female, n (%)	40 (27.2)	17 (12.9)	57 (20.4)
Mean body weight, kg (SD)	76.1 (15.9)	80.3 (18.7)	78.1 (17.4)
Mean age, years (SD)	41.4 (11.7)	42.3 (11.9)	41.8 (11.7)
Mean BCCL, mL/min (SD)	125 (31.3)	134 (35.8)	129 (33.7)
Mean BCRP, mg/dL (SD)	1.22 (1.35)	1.63 (1.73)	1.41 (1.55)
Race, n (%)			
White	116 (78.9)	107 (81.1)	223 (79.9)
Asian	31 (21.1)	24 (18.2)	55 (19.7)
Not available	0 (0.0)	1 (0.8)	1 (0.4)

BCCL = baseline creatinine clearance; BCRP = baseline C-reactive protein; N = number of evaluable patients; n = number of patients with characteristic; SD = standard deviation.

**Table 2.** Parameter and covariate parameter estimates for the full population PK model.

Parameter estimates for the final full model				
	Estimate (RSE%)	95% CI for estimate^a^	IIV (RSE%)	95% CI for IIV^a^
CL/F, L/hour	27.1 (2.41)	25.8, 28.5	28.2 (0.923)	21.4, 34.4
V/F, L	126 (2.81)	120, 134	36.6 (1.9)	25.6, 47.0
K_a_/hour^–1^	3.07 (10.2)	2.56, 3.74	NA	NA
Proportional error CV, TAD < 9 hours, %	60.2 (4.42)	54.8, 65.3	NA	NA
Proportional error CV, TAD ≥ 9 hours, %	69.6 (6.73)	60.4, 78.3	NA	NA
Covariance, CL/F-V/F	0.0760 (33.0)	0.0348, 0.131	NA	NA
Covariate parameter estimates				
PK parameter	Covariate		Estimate^b ^(RSE%)	95% CI^a^
CL/F	Age		–0.244 (33.7)	–0.419, –0.0989
CL/F	Sex: Female (vs. male)		0.0237 (177)	–0.0598, 0.107
CL/F	Race: Asian (vs. non-Asian)		–0.103 (31.7)	–0.164, –0.0385
CL/F	BCCL		0.233 (34.5)	0.0688, 0.377
CL/F	BCRP		–0.0185 (75.7)	–0.0447, 0.00881
V/F	Age		–0.230 (42.3)	–0.421, –0.0449
V/F	Body weight		0.574 (23.1)	0.306, 0.835

BCCL = baseline creatinine clearance; BCRP = baseline C-reactive protein; CI = confidence interval; CL/F = apparent oral clearance; CV = coefficient of variation; IIV = inter-individual variance; K_a_ = first-order absorption rate constant; NA = not available; PK = pharmacokinetics; RSE = relative standard error; TAD = time after dose; V/F = apparent volume of distribution. The derived elimination half-life was ~ 3 hours. ^a^Predicted by the bootstrap method. All 1,000 runs were minimized successfully. ^b^For continuous covariates, estimates were computed as power functions, normalized to the reference (approximate median) values in the dataset; for categorical covariates, estimates were computed as a fraction of the reference category, with one estimated coefficient for each category. Reference patient defined as: White, male, 78 kg, 40 years old, BCCL 126 mL/min, BCRP 0.851 mg/dL.

## Supplemental material

Supplemental material Figure S1
