# Supplementary material for: Population pharmacokinetics of tofacitinib in patients with active ankylosing spondylitis
Source: Int J Clin Pharmacol Ther. 2025 Dec 8;64(2):57–65. doi: 10.5414/CP204781 (PMC12825014; doi:10.5414/CP204781)
Supplement: Supplemental material [file intjclinpharmacol-64-057-S01.pdf]

Figure S1. Goodness-of-fit plots for the final full model: A) observed vs. predicted population concentration, stratified by dose, B) observed vs. individual predicted concentration by proportional error model (trough and non-trough data), and CWRES, stratified by C) study and D) dose.

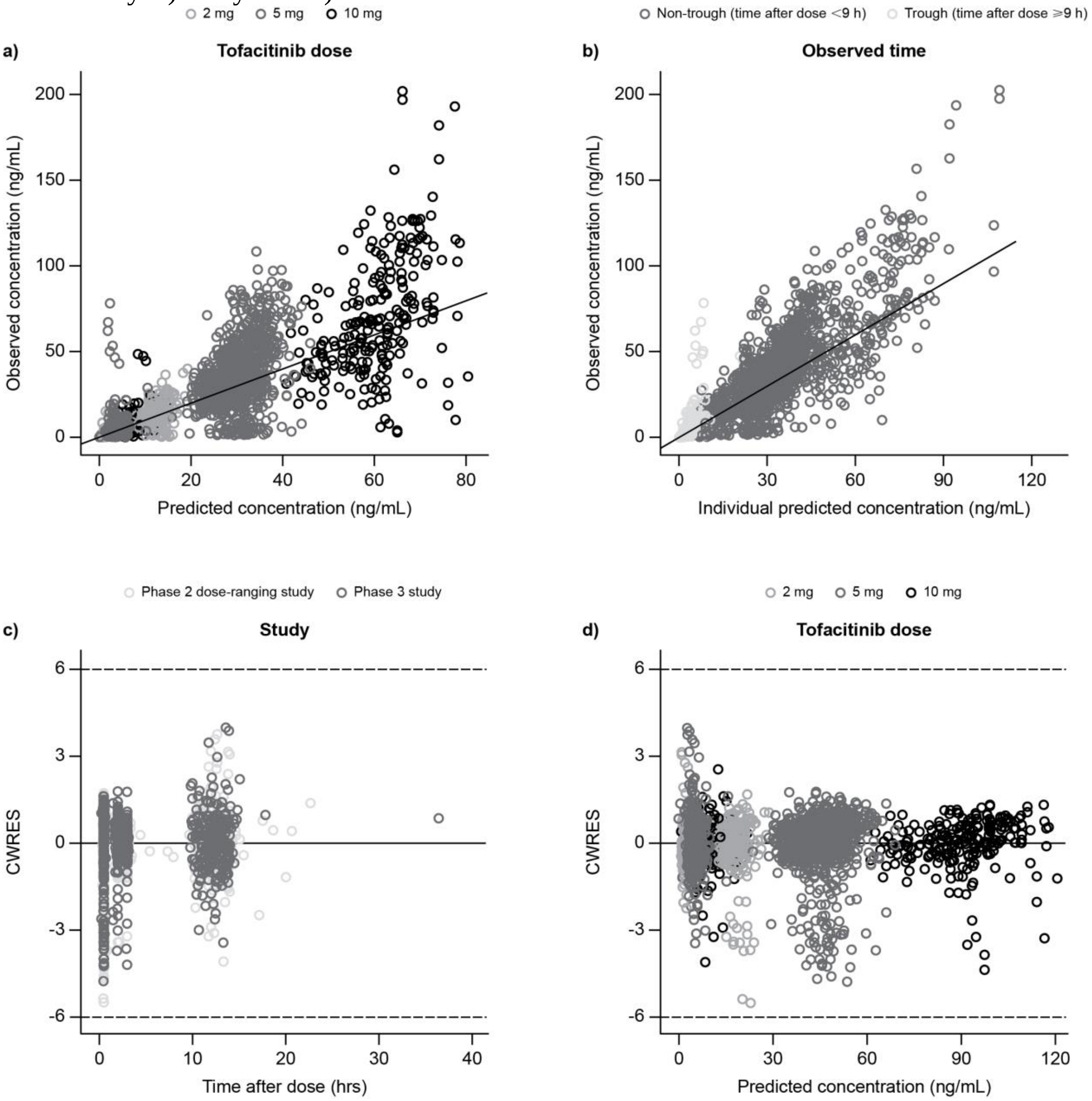

CWRES = conditional weighted residuals; h = hours; PK = pharmacokinetics.
The black solid line is a reference line of identity, and the black dashed line is the threshold for identifying outliers.
